# Distribution of Pathological Entities and Surgical Procedures Across Cervical Levels in Degenerative Cervical Myelopathy: An Exploratory Single-Center Analysis

**DOI:** 10.3390/jcm15218683

**Published:** 2026-11-07

**Authors:** Leonardo Anselmi, Gianpaolo Jannelli, Michele Da Broi, Aria Nouri, Nasser Bouhalassa, Federico Pessina, Enrico Tessitore

**Affiliations:** 1 Department of Neurosurgery, IRCCS Humanitas Research Hospital, Via Manzoni 56, Rozzano, 20089 Milan, Italy; 2 Division of Neurosurgery, Department of Clinical Neurosciences, Faculty of Medicine, Geneva University Hospitals and University of Geneva, Rue Gabrielle-Perret-Gentil 4, 1205 Geneva, Switzerland; 3 Department of Neurosurgery, Neurocenter of South Switzerland, Ente Ospedaliero Cantonale, Via Tesserete 46, 6900 Lugano, Switzerland; 4 Department of Biomedical Sciences, Humanitas University, Via Rita Levi Montalcini 4, Pieve Emanuele, 20072 Milan, Italy

**Keywords:** degenerative cervical myelopathy, surgical procedure selection, cervical level, anterior cervical discectomy and fusion, posterior fixation

## Abstract

**Background/Objectives:** Surgical planning in degenerative cervical myelopathy (DCM) considers the type of pathology, the number of levels involved, and cervical alignment and stability, but how the pathological entity, the operated level, and the procedure performed relate to one another remains unclear. This study described their distribution and associations in a consecutive surgical cohort. **Methods:** A total of 154 consecutive adults operated for DCM at a single center between 2013 and 2024 were studied retrospectively. Six non-mutually-exclusive pathological entities, five procedure types and every operated level (C2-C3 to T1-T2) were recorded, with combined procedures counted once. Associations were tested with Fisher–Freeman–Halton exact tests, with sensitivity analyses using a single dominant entity per patient and patient-level clustering, and a comparison of patients treated by ACDF alone versus any alternative strategy. **Results:** Procedure selection was related to the pathological entity (*p* < 0.001): isolated disc herniation was treated almost exclusively with ACDF (93.2%), whereas spondylolisthesis was the most heterogeneous, managed with posterior decompression and fixation (33.3%), ACCF (23.3%) or ACDF (23.3%); this persisted in the dominant-entity analysis (*p* = 0.002). The 46 patients (29.9%) not treated by ACDF alone were older, had more operated levels, worse baseline mJOA and more often circumferential compression (all *p* ≤ 0.005), whereas sagittal profile and cord and canal morphometry did not differ. Neither entities nor procedures were associated with the operated level across C3-C7 (*p* = 0.84 and *p* = 0.93); a nominally significant all-level result (*p* = 0.008) arose only from the sparsely operated terminal levels. **Conclusions:** Procedure selection in DCM was driven mainly by the underlying pathological entity, disease extent and compression geometry, rather than by the operated level or cervical alignment. No outcome data were analyzed, so these descriptive, hypothesis-generating findings require confirmation in larger multicenter cohorts with adequate representation of the terminal levels.

## 1. Introduction

Degenerative cervical myelopathy (DCM) is the most common cause of non-traumatic spinal cord dysfunction in adults, with an estimated prevalence of up to 2% [1,2,3]. DCM arises from chronic static and dynamic compression of the spinal cord by coexisting anterior and posterior degenerative changes, often accompanied by altered sagittal alignment [3,4,5]. Sustained compression triggers secondary injury, including ischemia and neuroinflammation, which typically presents with myelopathic signs such as gait disturbance, hand dysfunction, and impaired dexterity [5,6]. Left untreated, DCM tends to progress, and surgical decompression is the only treatment able to halt this decline and allow neurological recovery [2,6,7].

A wide range of surgical procedures is available, broadly divided into anterior approaches, such as anterior cervical discectomy and fusion (ACDF) and corpectomy and fusion (ACCF), and posterior approaches, comprising decompression alone or combined with instrumented fixation [8]. The choice between the two corridors has been studied extensively, and its main determinants are well recognized, including the number of levels involved, the morphology of compression, and sagittal alignment [8,9,10].

How the underlying pathology, its extent, and the level involved translate into the specific procedure actually performed has been less extensively explored. Large multicenter studies have described how often each degenerative entity occurs and where the cord is most often compressed [11], and MRI-based pathological features have been related to the choice between anterior and posterior approaches [12]. What remains unquantified is how the pathological entity, the operated level, and the procedure performed are distributed and associated in a consecutive surgical cohort. The objective of this exploratory single-center study was to describe these patterns, which may complement existing approach-based frameworks and inform a more pathology-driven model of surgical planning.

## 2. Materials and Methods

### 2.1. Study Design and Population

The present study is a retrospective analysis of data prospectively collected within a study approved by the Ethics Committee of Geneva University Hospitals (protocol 11-194; NAC 11-067). Consecutive adult patients (≥18 years) surgically treated for degenerative cervical myelopathy (DCM) between 2013 and 2024 were included. Inclusion criteria comprised: (1) a clinical diagnosis of DCM, defined as objective signs of spinal cord dysfunction including upper motor neuron signs, gait disturbance, hand dysfunction, or sphincter involvement, in the absence of an alternative neurological diagnosis; (2) radiological confirmation of cervical spinal cord compression on MRI, with or without intramedullary T2 signal changes; (3) surgical decompression performed via an anterior, posterior, or combined approach; and (4) complete data on the pathological entity, the operated levels, and the procedure performed. Patients with isolated radiculopathy, non-degenerative etiologies (trauma, infection, tumor), or previous cervical spine surgery were excluded, as were patients for whom informed consent for the use of clinical data was not available.

### 2.2. Classification of Pathology, Procedure, and Level

For each patient, the compressive pathology was classified into six non-mutually-exclusive entities: isolated disc herniation, multilevel disc disease, ligamentum flavum hypertrophy, spondylolisthesis (anterior or posterior translation of a vertebral body relative to the adjacent level on neutral sagittal imaging), congenital stenosis (spinal cord occupation ratio, SCOR, of 70% or greater, calculated from the anteroposterior diameters of the spinal cord and spinal canal measured at non-compressed levels above and below the region of interest [13]), and ossification of the posterior longitudinal ligament (OPLL); coexisting entities were each recorded separately. Surgical procedures were grouped into five categories: ACDF, ACCF, posterior decompression alone, posterior decompression with instrumented fixation, and combined procedures, defined as two or more distinct techniques performed during the same operation. To avoid double counting, combined procedures were counted once as a single category rather than assigned to their component techniques. Because a combined procedure represents a distinct and more extensive strategy than a stand-alone ACDF, patients treated with a combined procedure were grouped with those not treated by isolated ACDF in the subgroup comparison, even though all of them included an ACDF component. Each operated level was recorded individually, from C2-C3 to T1-T2. For the sensitivity analysis, each patient was additionally assigned a single dominant entity according to a pre-specified hierarchy reflecting structural severity: OPLL, spondylolisthesis, congenital stenosis, multilevel disc disease, ligamentum flavum hypertrophy, isolated disc herniation.

### 2.3. Clinical and Radiological Variables

Baseline neurological function was graded with the modified Japanese Orthopaedic Association (mJOA) score. Morphometric variables were measured on preoperative MRI at the level of maximal cord compression and at the nearest non-compressed level above and below. Cord compromise was defined as 1 − (d_i_/mean of d above and d below), where d is the anteroposterior diameter of the spinal cord, and canal compromise analogously from the anteroposterior diameter of the spinal canal; the spinal cord occupation ratio (SCOR) was the mean cord diameter divided by the mean canal diameter at the non-compressed levels [13]. The direction of compression was classified as purely ventral, purely dorsal, or circumferential. Cervical sagittal profile was assessed separately on preoperative lateral cervical radiographs, using the C2–C7 Cobb angle, and classified according to a predefined institutional criterion as kyphotic (<10°), normal (10–40°) or hyperlordotic (>40°).

### 2.4. Statistical Analysis

Continuous variables are reported as mean ± standard deviation or median with interquartile range (IQR). The association between procedure and pathology was assessed with the Fisher–Freeman–Halton exact test and, because entities were non-mutually-exclusive, repeated using the single dominant entity per patient, so that each patient contributed once (Monte Carlo exact test). To characterize the patients in whom the default anterior strategy was not used, those treated by ACDF alone were compared with those managed by any other strategy (ACCF, posterior decompression with or without fixation, or a combined procedure) on demographic, clinical and radiological variables, using the Mann–Whitney U test for continuous and the Fisher exact or chi-square test for categorical variables. In the level-based analyses, each patient contributes one entry per operated level, so totals exceed the number of patients; these tables are therefore expressed as operated-level entries, the number of contributing patients is given for each level, and all level-based models were refitted with cluster-robust standard errors grouped by patient. The C3-C7 analysis, which excludes the sparsely operated terminal levels, was pre-specified as the primary level-based analysis. A rostral-to-caudal gradient was tested with the Cochran–Armitage trend test, with the direction and magnitude of each relationship summarized by ordinal logistic regression as an odds ratio (OR) per caudal level (95% confidence interval, CI); a global Fisher–Freeman–Halton test was also reported and was repeated as a sensitivity analysis with the patient, rather than the individual operated-level entry, as the unit of permutation. Analyses were performed in Python (version 3.12); *p* < 0.05 was considered significant.

## 3. Results

### 3.1. Patient Population

Between January 2013 and December 2024, 188 consecutive patients surgically treated for degenerative cervical myelopathy were screened. Thirty-four were excluded because informed consent for the use of their clinical data was not available, leaving 154 patients for analysis. The mean age was 58.6 ± 11.4 years, with a balanced sex distribution (52.6% male). Multilevel disc disease was the most frequent pathological entity (58.4%), followed by isolated disc herniation (38.3%) and ligamentum flavum hypertrophy (33.1%), and entities frequently co-occurred. ACDF was the most frequently performed procedure (70.1%). Full baseline characteristics are summarized in Table 1.

### 3.2. Procedure Selection by Pathological Entity

**Figure 1:**
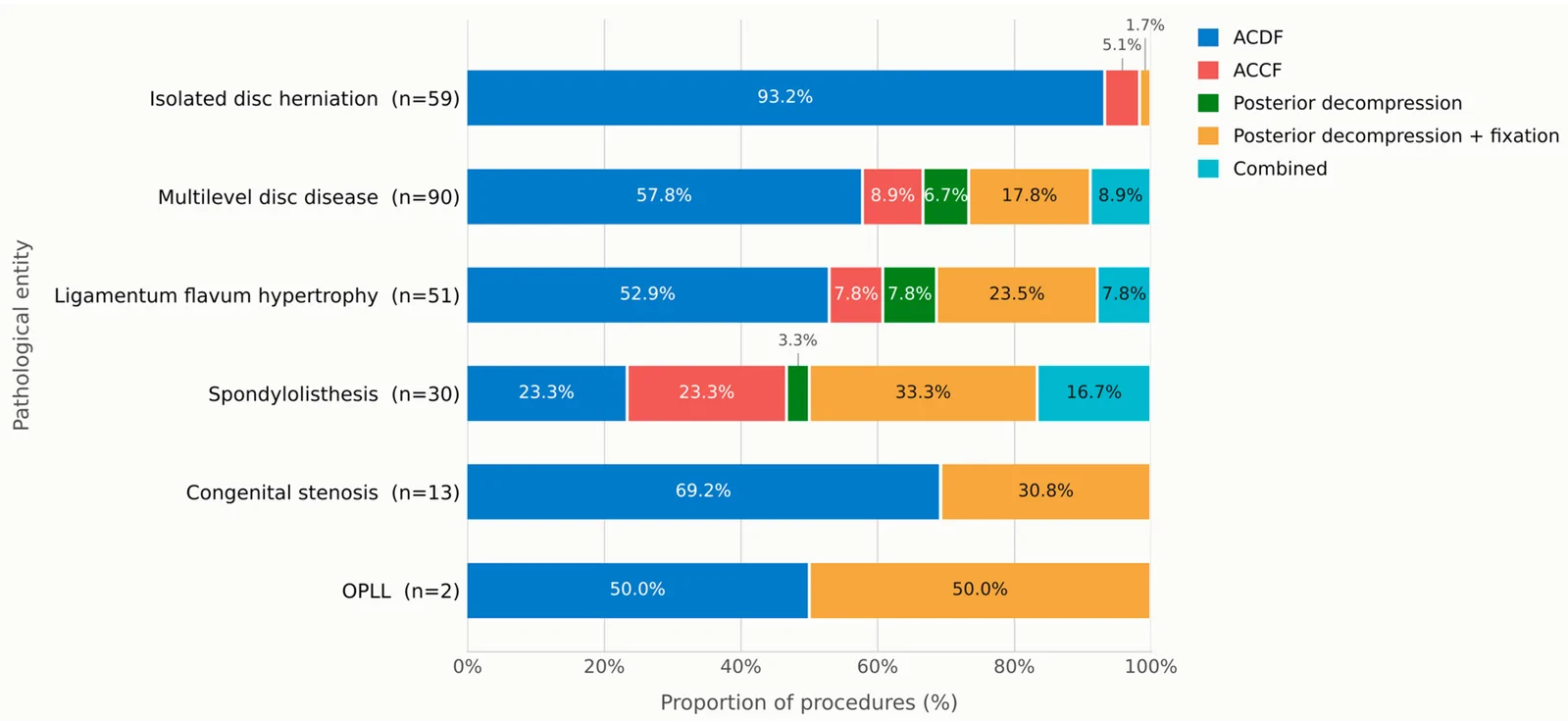
Distribution of surgical procedures within each pathological entity. Bars are scaled to 100% of the procedures performed for that entity; *n* is the number of patients in whom the entity was recorded. Entities are non-mutually-exclusive. ACDF, anterior cervical discectomy and fusion; ACCF, anterior cervical corpectomy and fusion; OPLL, ossification of the posterior longitudinal ligament.

Procedure selection differed significantly across pathological categories (*p* < 0.001). Isolated disc herniation was managed almost exclusively with ACDF (93.2%). Multilevel disc disease was treated mainly with ACDF (57.8%), followed by posterior decompression with fixation (17.8%). Ligamentum flavum hypertrophy showed a similar pattern (ACDF 52.9%, posterior decompression with fixation 23.5%). Spondylolisthesis had the most varied distribution, most often managed with posterior decompression with fixation (33.3%), ACCF (23.3%), or ACDF (23.3%). Congenital stenosis was treated with ACDF (69.2%) or posterior decompression with fixation (30.8%). Only two patients had OPLL, one treated with ACDF and one with posterior decompression with fixation; given this number, no entity-specific inference is drawn for OPLL, and findings for congenital stenosis (*n* = 13) are likewise reported descriptively (Table 2 and Figure 1).

In the sensitivity analysis restricted to a single dominant entity per patient, which removes the overlap between co-occurring entities, the association between pathology and procedure was confirmed (Monte Carlo exact *p* = 0.002). Spondylolisthesis remained the clear outlier, managed with ACDF in 23.3% of cases compared with 72.3% to 94.4% across all other dominant-entity groups (Table 3).

### 3.3. Patients Managed Without Isolated ACDF

A total of 108 patients (70.1%) were treated by ACDF alone and 46 (29.9%) by an alternative strategy, comprising ACCF in 12, posterior decompression with fixation in 20, posterior decompression alone in 6 and a combined procedure in 8. Each combined procedure included an anterior cervical discectomy and fusion: five were ACDF with ACCF, two ACDF with ACCF and posterior decompression with fixation, and one ACDF with posterior decompression with fixation; three therefore involved both an anterior and a posterior approach.

Patients managed without isolated ACDF were older (median 65 years, IQR 58–74, vs. 54, IQR 47–64; *p* < 0.001), were operated at a greater number of levels (3, IQR 2–4, vs. 1, IQR 1–2; *p* < 0.001), presented with worse baseline neurological function (mJOA 13, IQR 11–15, vs. 15, IQR 12–15; *p* = 0.005), and more frequently had circumferential rather than purely ventral cord compression (56.5% vs. 26.2%; *p* = 0.001). Spondylolisthesis was present in 50.0% of this group versus 6.5% (*p* < 0.001) and multilevel disc disease in 82.6% versus 48.1% (*p* < 0.001). Sagittal profile (kyphotic in 53.7% vs. 44.0%; *p* = 0.36), spinal cord occupation ratio (*p* = 0.64), cord compromise (*p* = 0.33), canal compromise (*p* = 0.48), body mass index (*p* = 0.76), and sex (*p* = 0.22) did not differ (Table 4).

### 3.4. Pathology Distribution by Operated Level

Pathological entities were recorded at each operated level from C2-C3 to T1-T2, giving 317 operated-level entries across the 154 patients. Multilevel disc disease was the most frequent entity at every level from C3-C4 to C6-C7 (35.9–47.3%), while ligamentum flavum hypertrophy (21.2–25.2%), isolated disc herniation (8.8–16.5%), spondylolisthesis (11.2–16.5%), and congenital stenosis (4.3–6.5%) occurred in comparable proportions across levels; OPLL was limited to a few upper and mid-cervical entries.

On directional analysis, two entities tended to be more frequent at rostral levels, both as non-significant trends: spondylolisthesis (OR 0.79 per caudal level, 95% CI 0.61–1.04) and ligamentum flavum hypertrophy (OR 0.85, 95% CI 0.67–1.07). OPLL pointed in the same direction with the largest estimate (OR 0.57) but the widest interval (0.20–1.61), reflecting its rarity in this cohort. Multilevel disc disease was the only entity trending caudally (OR 1.11, 95% CI 0.85–1.43), whereas isolated disc herniation (OR 0.98) and congenital stenosis (OR 0.94) were evenly distributed.

**Figure 2:**
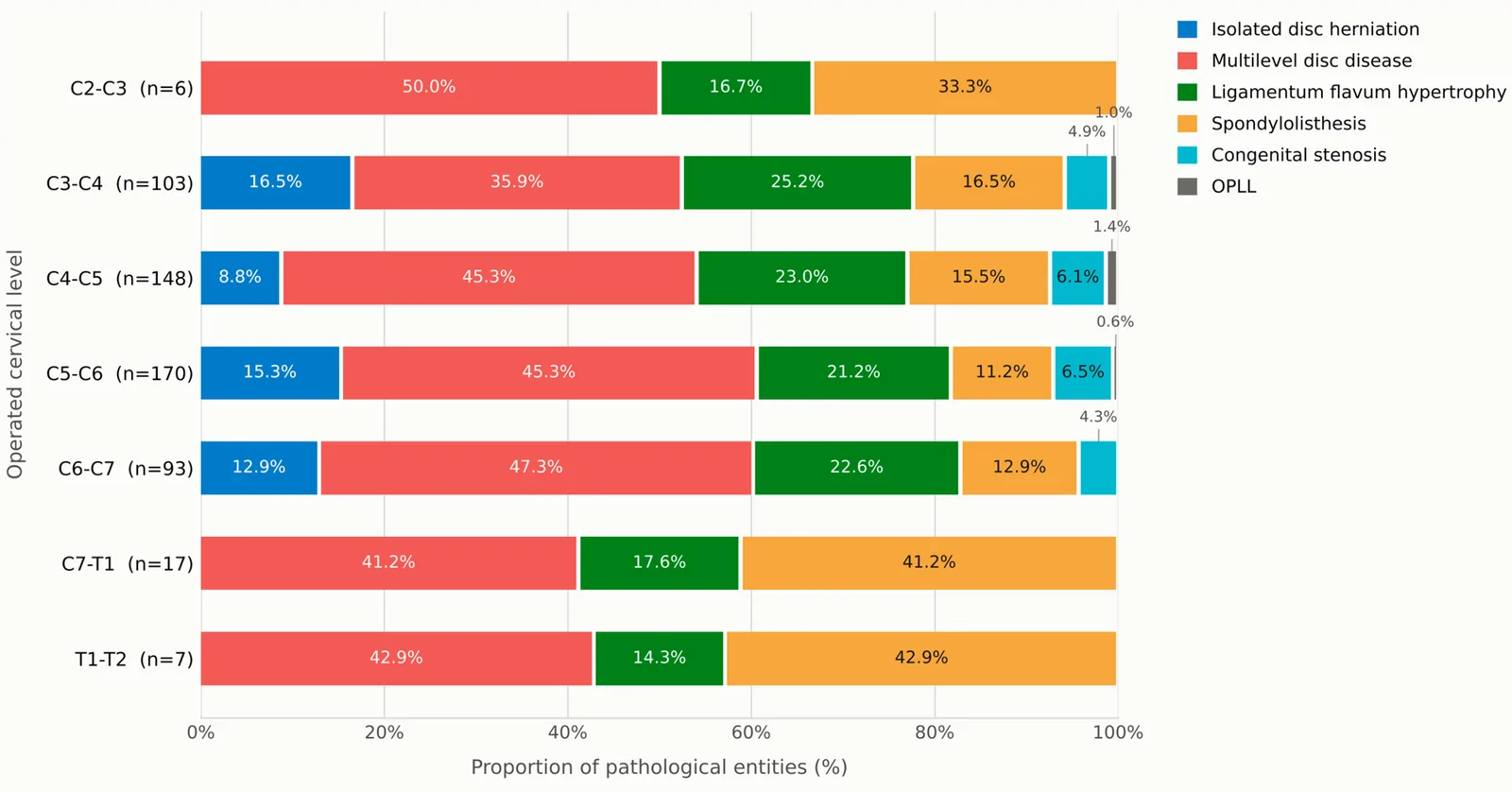
Distribution of pathological entities at each operated cervical level. Bars are scaled to 100% of the entities recorded at that level; *n* is the number of entity records, which exceeds the number of operated levels because entities are non-mutually-exclusive and frequently co-occurred at the same level. The terminal levels (C2-C3, C7-T1, T1-T2) rest on few records, and their proportions should be interpreted accordingly. OPLL, ossification of the posterior longitudinal ligament.

The global Fisher–Freeman–Halton test was non-significant across the C3-C7 levels (*p* = 0.84) and did not reach significance when the terminal levels (C2-C3, C7-T1, T1-T2) were included (C2-T2, *p* = 0.45). The patient-level permutation analysis gave *p* = 0.13 across C3-C7 and *p* = 0.041 across all operated levels, the latter arising from the terminal levels. The entities recorded at those segments were mainly multilevel disc disease and spondylolisthesis, both also common throughout the cervical spine (Table 5, Figure 2).

### 3.5. Procedure Selection by Operated Level

**Figure 3:**
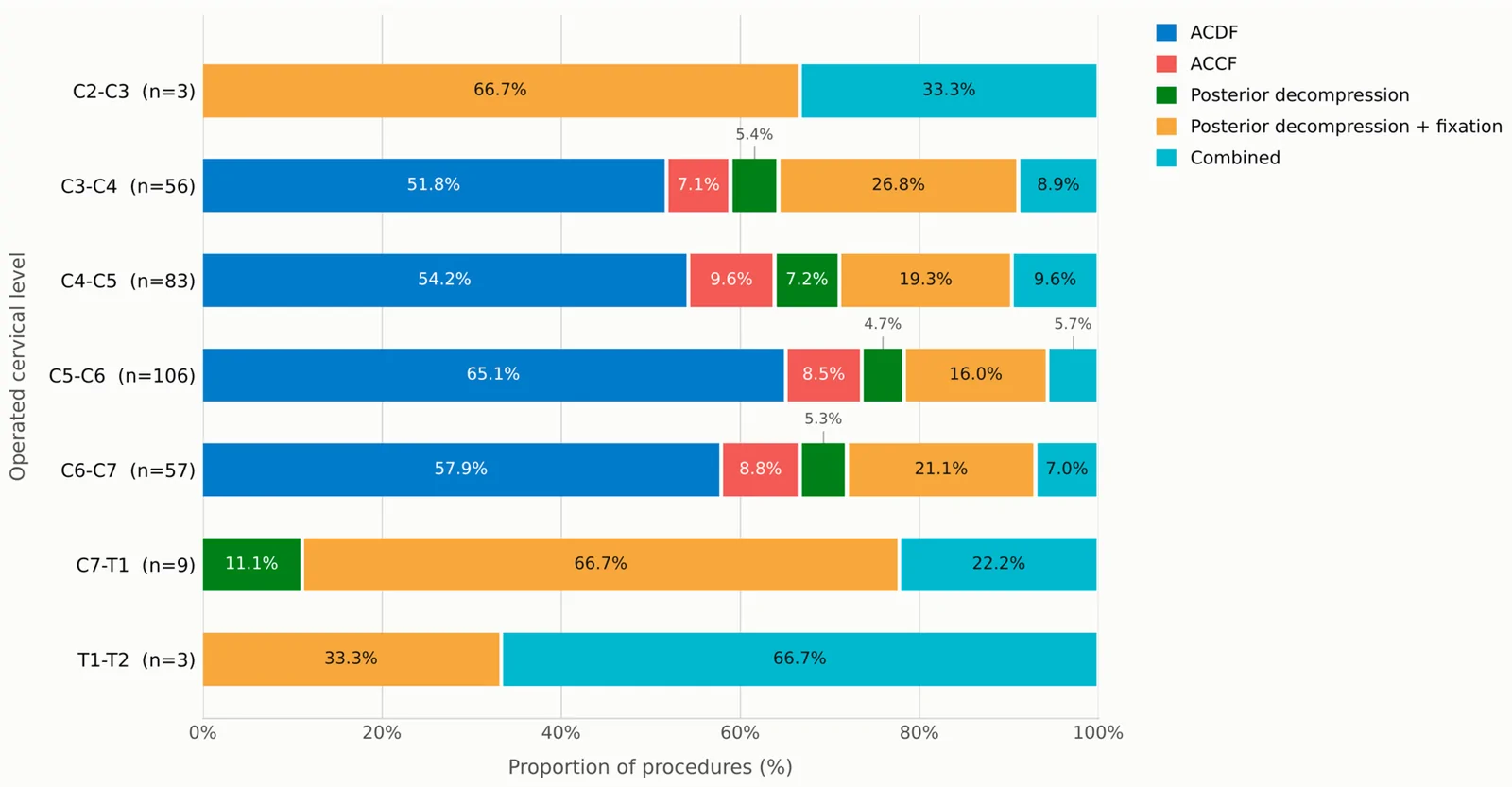
Distribution of surgical procedures at each operated cervical level. Bars are scaled to 100% of the procedures performed at that level; *n* is the number of operated-level entries (317 in total). No ACDF was performed at the terminal levels (C2-C3, C7-T1, T1-T2), which rest on three to nine entries each. ACDF, anterior cervical discectomy and fusion; ACCF, anterior cervical corpectomy and fusion.

Across the same 317 operated-level entries, ACDF was the predominant procedure at all levels from C3-C4 to C6-C7 (51.8–65.1%), followed by posterior decompression with fixation (16.0–26.8%). At the terminal levels (C2-C3, C7-T1, T1-T2), where only a few procedures were performed, no ACDF was recorded, and treatment consisted almost exclusively of posterior decompression with fixation or combined procedures (Table 6, Figure 3).

On directional analysis, ACDF was the only procedure trending toward caudal levels (OR 1.15 per caudal level, 95% CI 0.92–1.45), while posterior decompression with fixation (OR 0.87, 95% CI 0.66–1.16) and combined procedures (OR 0.85, 95% CI 0.55–1.30) trended rostrally, and ACCF (OR 1.04) and posterior decompression alone (OR 0.93) were essentially flat. All confidence intervals crossed 1, and refitting the models with cluster-robust standard errors grouped by patient left every estimate substantially unchanged.

In the pre-specified primary analysis, restricted to C3-C7, procedure selection showed no association with the operated level (global Fisher–Freeman–Halton *p* = 0.93), whereas across all operated levels the test was nominally significant (C2-T2, *p* = 0.008). This difference came entirely from the terminal levels. At C2-C3, C7-T1, and T1-T2, no anterior procedure of any kind was performed, and treatment consisted almost exclusively of posterior decompression with fixation or of a combined construct, although each of these segments rests on only three to nine operated-level entries. Repeating both tests with the patient rather than the individual entry as the unit of permutation gave the same result (C3-C7 *p* = 0.57; C2-T2 *p* = 0.005).

## 4. Discussion

Surgical planning in DCM takes into account several parameters, such as the type of pathology, the number of levels involved, and cervical alignment and stability, with different surgical techniques available to address these situations [8,9]. Several previous studies have investigated these factors individually: multicenter cohorts have described the prevalence and spectrum of the pathological entities [11], including how conditions such as spondylolisthesis affect clinical presentation and outcome [14]; anterior and posterior approaches yield comparable neurological outcomes, with recovery depending mainly on baseline severity rather than on the surgical corridor [12,15,16]; and decision-making frameworks and current AO Spine RECODE-DCM priorities emphasize the individualization of surgical treatment [9,17,18]. However, how the pathological entity, the operated level, and the chosen procedure relate to one another, and thereby guide surgical decision-making, has not been fully clarified.

In the present cohort, we found a significant association between the surgical technique and the pathological entity underlying DCM (*p* < 0.001). Myelopathic patients with a diagnosis of isolated disc herniation were treated almost exclusively with ACDF (93.2%). This is consistent with standard clinical practice and with the WFNS Spine Committee recommendations, which identify anterior pathology with compression involving fewer than three levels as the primary indication for ACDF [19]. This technique provides direct spinal cord decompression with excellent restoration of segmental height and lordosis, fusion rates exceeding 90% in modern series [9,20], a low complication profile with limited approach-related morbidity [21,22], and sustained neurological recovery at long-term follow-up [23]. These findings support the favorable risk–benefit profile that has established ACDF as the reference technique for single-level cervical disc pathology [9,23]. Because several entities co-occurred in the same patient, this association was re-examined after assigning each patient a single dominant entity; it remained significant (*p* = 0.002), indicating that the pattern is not an artifact of overlapping categories.

On the other hand, the management of spondylolisthesis was considerably more heterogeneous. Degenerative cervical spondylolisthesis is rarely an isolated segmental disorder and is typically associated with concomitant multilevel degenerative changes, including multilevel disc disease, facet arthrosis, and ligamentous hypertrophy, reflecting a diffuse pathophysiological cascade that spans multiple cervical segments [24,25]. Consistently, spondylolisthesis patients in this cohort rarely presented with single-level disease and were operated on at a significantly greater number of levels than patients with isolated disc herniation (median 2, IQR 2–4, *n* = 30, vs. 1, IQR 1–1, *n* = 59; Mann–Whitney U = 1492, *p* < 0.001). When multilevel decompression is required, extending ACDF carries increasing risks of dysphagia, pseudoarthrosis, and construct-related complications [21,26], and posterior decompression with instrumented fixation is generally preferred when three or more levels are involved [19,27]. Accordingly, only 23.3% of the spondylolisthesis cases were managed with a stand-alone ACDF, while the remainder required a more extensive construct, most often posterior decompression with fixation (33.3%) or ACCF (23.3%), and less frequently a combined procedure (16.7%) or posterior decompression alone (3.3%). A posterior approach was involved in 43.3% overall. ACCF was used when a single-level discectomy was inadequate to achieve sufficient decompression [19]. This distribution confirms that the multilevel nature of degenerative spondylolisthesis demands more extensive surgical strategies than focal single-level pathology [8,19,27].

The patients in whom the default anterior strategy was not used represent the most informative subgroup of this cohort. They were older, more extensively affected and more severely myelopathic at baseline; half of them had spondylolisthesis, and circumferential compression was more frequent, a combination in which a single anterior construct addresses neither the dorsal component of compression nor the segmental instability. The parameters that did not differ are equally relevant. Preoperative sagittal profile was comparable between the two groups, as were the spinal cord occupation ratio and the canal and cord compromise indices. These findings do not compete with the pathological classification, because the entities used here are themselves defined in anatomical and mechanical terms. Isolated disc herniation and multilevel disc disease differ in how far the disease extends. Spondylolisthesis means segmental instability. Congenital stenosis describes a narrow canal along its whole length rather than a single compressed level. Disease extent, instability and the direction of compression are therefore what the entity already contains, and the association between pathology and procedure described above can be understood as working through them. Age fits the same picture, since it was unrelated to the number of operated levels but was clearly higher in patients with spondylolisthesis. The multiparametric frameworks used in current practice [9,17,18] and the pathology-driven pattern seen here are thus two ways of describing the same decision, with the entity acting as a shorthand for the features that determine what the operation must achieve. The one thing it does not capture is where the compression sits along the cervical spine, which is also the only dimension unrelated to procedure selection.

In the present study, the distribution of pathological entities and surgical procedures across operated levels was also assessed. Neither analysis reached statistical significance across C3-C7, and no clear rostro-caudal gradient emerged. However, this finding should be interpreted with caution rather than as definitive evidence of no association. The terminal levels (C2–C3, C7–T1, and T1–T2) were underrepresented in this cohort, and the small number of cases at these segments may have been insufficient to detect a real effect. The cervical spine is not biomechanically uniform: segmental mobility, loading patterns, and sagittal alignment vary along the subaxial spine, particularly at the cervicothoracic junction where a mobile segment transitions into a rigid one [28,29]. These mechanical differences may influence both the type of degenerative pathology that develops at each level and the surgical technique best suited to address it. This is most plausible at the cervicothoracic junction, where the transition from a mobile to a rigid segment concentrates mechanical stress and may favor instrumented posterior or combined constructs over a stand-alone anterior procedure; consistent with this, no anterior procedure of any kind was performed at C2-C3, C7-T1 or T1-T2 in the present cohort, and fourteen of the fifteen procedures recorded at those segments included instrumented fusion. Repeating the global tests with the patient as the unit of permutation, rather than the individual operated-level entry, did not change this interpretation. Neither pathology nor procedure was associated with the operated level across C3-C7, while both reached nominal significance once the terminal levels were included, confirming that the signal arises from those segments and not from treating repeated entries as independent. Larger multicenter cohorts with adequate representation of the terminal levels would be needed to determine whether a level-dependent component exists and could complement the predominantly pathology-driven pattern observed in this study.

## 5. Limitations

Several limitations should be acknowledged. The design was retrospective and single-center, and procedures were distributed as they are in routine practice rather than by allocation, so the pattern described here reflects institution-specific practice and may not generalize. Isolated disc herniation and ACDF predominated, which alone may partly explain the uniform distribution observed across levels. Because several entities often coexisted in the same patient, the distributional analyses are not fully independent, and procedure choice cannot be attributed to a single entity. OPLL, congenital stenosis and the extreme cervical levels were represented by few cases, which limits statistical power and makes these analyses descriptive rather than confirmatory. Sagittal alignment and the morphometric indices of canal and cord narrowing were examined only in the comparison of patients treated by ACDF alone with those managed otherwise, where neither discriminated between the groups and were not modeled as determinants of procedure choice across the whole cohort. The reasoning behind each individual surgical decision, including surgeon preference, was not recorded prospectively and could not be assessed. In the analysis dataset, each entity was attributed to every level operated in that patient rather than to the level at which it was located, so the level-based tables show the entities present in patients operated at a given level. Finally, no postoperative outcome data are reported. The present study therefore describes how procedures were distributed and cannot speak to their appropriateness or effectiveness.

## 6. Conclusions

In this consecutive single-center cohort, the choice of surgical procedure in DCM was significantly related to the underlying pathological entity. In the pre-specified analysis restricted to C3-C7, by contrast, neither the procedures nor the pathological entities were related to the operated level. A nominally significant result emerged only once the sparsely operated terminal levels were included, and the small number of cases at those segments means that a level-dependent component can be neither demonstrated nor excluded. Cervical alignment and the morphometric indices of compression did not distinguish patients managed by ACDF alone from those requiring an alternative strategy, whereas disease extent and the direction of compression did. These findings describe practice patterns only and carry no implication as to the appropriateness or effectiveness of the procedures analyzed, since no outcome data were examined. Larger multicenter cohorts, with adequate representation of the junctional segments and with clinical outcomes, will be needed to establish whether the operated level contributes to surgical decision-making and whether modeling these parameters jointly can refine individualized surgical planning.

## Figures and Tables

**Table 1:** Baseline characteristics of the study cohort (*n* = 154).

Characteristic	Value
Age, years—mean ± SD	58.6 ± 11.4
Age, years—median (IQR)	58 (51–68)
Male sex	81 (52.6%)
Female sex	73 (47.4%)
Body mass index, kg/m^2^—mean ± SD	26.5 ± 4.6
Isolated disc herniation	59 (38.3%)
Multilevel disc disease	90 (58.4%)
Ligamentum flavum hypertrophy	51 (33.1%)
Spondylolisthesis	30 (19.5%)
Congenital stenosis	13 (8.4%)
OPLL	2 (1.3%)
ACDF	108 (70.1%)
ACCF	12 (7.8%)
Posterior decompression alone	6 (3.9%)
Posterior decompression with fixation	20 (13.0%)
Combined procedures	8 (5.2%)

Data are *n* (%) unless otherwise stated. Pathological entities are non-mutually-exclusive and frequently co-occurred. SD, standard deviation; IQR, interquartile range; ACDF, anterior cervical discectomy and fusion; ACCF, anterior cervical corpectomy and fusion; OPLL, ossification of the posterior longitudinal ligament.

**Table 2:** Surgical procedures by pathological entity (*n* = 154).

Pathology	ACDF	ACCF	Post Dec	Post Dec + Fix	Combined	Total
Isolated disc herniation	55 (93.2%)	3 (5.1%)	0 (0.0%)	1 (1.7%)	0 (0.0%)	59
Multilevel disc disease	52 (57.8%)	8 (8.9%)	6 (6.7%)	16 (17.8%)	8 (8.9%)	90
Ligamentum flavum hypertrophy	27 (52.9%)	4 (7.8%)	4 (7.8%)	12 (23.5%)	4 (7.8%)	51
Spondylolisthesis	7 (23.3%)	7 (23.3%)	1 (3.3%)	10 (33.3%)	5 (16.7%)	30
Congenital stenosis	9 (69.2%)	0 (0.0%)	0 (0.0%)	4 (30.8%)	0 (0.0%)	13
OPLL	1 (50.0%)	0 (0.0%)	0 (0.0%)	1 (50.0%)	0 (0.0%)	2

Data are *n* (%). Fisher–Freeman–Halton exact test, *p* < 0.001. Pathological entities are non-mutually-exclusive. Post Dec, posterior decompression alone; Post Dec + Fix, posterior decompression with instrumented fixation; ACDF, anterior cervical discectomy and fusion; ACCF, anterior cervical corpectomy and fusion; OPLL, ossification of the posterior longitudinal ligament.

**Table 3:** Sensitivity analysis: Surgical procedures by single dominant pathological entity (*n* = 154).

Dominant Entity	ACDF	ACCF	Post Dec	Post Dec + Fix	Combined	Total
Spondylolisthesis	7 (23.3%)	7 (23.3%)	1 (3.3%)	10 (33.3%)	5 (16.7%)	30
Multilevel disc disease	47 (72.3%)	3 (4.6%)	5 (7.7%)	7 (10.8%)	3 (4.6%)	65
Congenital stenosis	8 (88.9%)	0 (0.0%)	0 (0.0%)	1 (11.1%)	0 (0.0%)	9
Ligamentum flavum hypertrophy	11 (91.7%)	1 (8.3%)	0 (0.0%)	0 (0.0%)	0 (0.0%)	12
Isolated disc herniation	34 (94.4%)	1 (2.8%)	0 (0.0%)	1 (2.8%)	0 (0.0%)	36
OPLL	1 (50.0%)	0 (0.0%)	0 (0.0%)	1 (50.0%)	0 (0.0%)	2

Data are *n* (%). Each patient contributes once. Entities are ordered by the pre-specified hierarchy (see Methods section). Monte Carlo exact test, *p* = 0.002. Post Dec, posterior decompression alone; Post Dec + Fix, posterior decompression with instrumented fixation.

**Table 4:** Comparison of patients treated by ACDF alone and by any alternative strategy.

Variable	ACDF Alone (*n* = 108)	Other Strategy (*n* = 46)	*p*
Age, years	54 (47–64)	65 (58–74)	<0.001
Male sex	53 (49.1%)	28 (60.9%)	0.218
Body mass index, kg/m^2^	26.1 (23.4–29.2)	26.1 (22.6–29.4)	0.755
Operated levels, *n*	1 (1–2)	3 (2–4)	<0.001
Baseline mJOA	15 (12–15)	13 (11–15)	0.005
SCOR	0.61 (0.56–0.65)	0.60 (0.56–0.65)	0.636
Cord compromise	0.29 (0.17–0.39)	0.30 (0.23–0.40)	0.325
Canal compromise	0.52 (0.39–0.59)	0.51 (0.42–0.60)	0.476
Sagittal profile—kyphotic	44 (44.0%)	22 (53.7%)	0.357
Sagittal profile—normal	53 (53.0%)	19 (46.3%)	
Sagittal profile—hyperlordotic	3 (3.0%)	0 (0.0%)	
Compression—purely ventral	78 (72.9%)	20 (43.5%)	0.001
Compression—purely dorsal	1 (0.9%)	0 (0.0%)	
Compression—circumferential	28 (26.2%)	26 (56.5%)	
Isolated disc herniation	55 (50.9%)	4 (8.7%)	<0.001
Multilevel disc disease	52 (48.1%)	38 (82.6%)	<0.001
Ligamentum flavum hypertrophy	27 (25.0%)	24 (52.2%)	0.001
Spondylolisthesis	7 (6.5%)	23 (50.0%)	<0.001
Congenital stenosis	9 (8.3%)	4 (8.7%)	1.000
OPLL	1 (0.9%)	1 (2.2%)	0.510

Data are median (IQR) or *n* (%). Mann–Whitney U test for continuous variables; Fisher exact or chi-square test for categorical variables. mJOA, modified Japanese Orthopaedic Association score; SCOR, spinal cord occupation ratio.

**Table 5:** Pathological entities by operated cervical level.

Level	Isolated Disc	Multilevel Disc	LF Hyper.	DCS	Congenital Stenosis	OPLL	Entity Records (*n*)	Patients Operated (*n*)
C2-C3	0 (0.0%)	3 (50.0%)	1 (16.7%)	2 (33.3%)	0 (0.0%)	0 (0.0%)	6	3
C3-C4	17 (16.5%)	37 (35.9%)	26 (25.2%)	17 (16.5%)	5 (4.9%)	1 (1.0%)	103	56
C4-C5	13 (8.8%)	67 (45.3%)	34 (23.0%)	23 (15.5%)	9 (6.1%)	2 (1.4%)	148	83
C5-C6	26 (15.3%)	77 (45.3%)	36 (21.2%)	19 (11.2%)	11 (6.5%)	1 (0.6%)	170	106
C6-C7	12 (12.9%)	44 (47.3%)	21 (22.6%)	12 (12.9%)	4 (4.3%)	0 (0.0%)	93	57
C7-T1	0 (0.0%)	7 (41.2%)	3 (17.6%)	7 (41.2%)	0 (0.0%)	0 (0.0%)	17	9
T1-T2	0 (0.0%)	3 (42.9%)	1 (14.3%)	3 (42.9%)	0 (0.0%)	0 (0.0%)	7	3

All 95% CIs crossed 1. Global Fisher–Freeman–Halton test non-significant across the C3-C7 levels (*p* = 0.84) and across all operated levels (C2-T2, *p* = 0.45). Entity records are the pathological entities recorded in the patients operated at each level; because entities co-occur, they exceed the number of patients. The 317 operated-level entries derive from 154 patients. Patient-level permutation analysis: C3-C7 *p* = 0.13; C2-T2 *p* = 0.041. LF hyper., ligamentum flavum hypertrophy; DCS, degenerative cervical spondylolisthesis; OPLL, ossification of the posterior longitudinal ligament.

**Table 6:** Surgical procedures by operated cervical level.

Level	ACDF	ACCF	Post Dec	Post Dec + Fix	Combined	Patients Operated (*n*)
C2-C3	0 (0.0%)	0 (0.0%)	0 (0.0%)	2 (66.7%)	1 (33.3%)	3
C3-C4	29 (51.8%)	4 (7.1%)	3 (5.4%)	15 (26.8%)	5 (8.9%)	56
C4-C5	45 (54.2%)	8 (9.6%)	6 (7.2%)	16 (19.3%)	8 (9.6%)	83
C5-C6	69 (65.1%)	9 (8.5%)	5 (4.7%)	17 (16.0%)	6 (5.7%)	106
C6-C7	33 (57.9%)	5 (8.8%)	3 (5.3%)	12 (21.1%)	4 (7.0%)	57
C7-T1	0 (0.0%)	0 (0.0%)	1 (11.1%)	6 (66.7%)	2 (22.2%)	9
T1-T2	0 (0.0%)	0 (0.0%)	0 (0.0%)	1 (33.3%)	2 (66.7%)	3

All 95% CIs for the odds ratios per caudal level crossed 1. Global Fisher–Freeman–Halton test non-significant across the C3-C7 levels (*p* = 0.93) but nominally significant across all operated levels (C2-T2, *p* = 0.008), the latter attributable to the low-frequency terminal levels (C2-C3, C7-T1, T1-T2). Each patient contributed one procedure per operated level, so the number of patients operated at a level equals the number of operated-level entries at that level. The 317 entries derive from 154 patients. Patient-level permutation analysis: C3-C7 *p* = 0.57; C2-T2 *p* = 0.005. Post Dec, posterior decompression alone; Post Dec + Fix, posterior decompression with instrumented fixation.

## Data Availability

All relevant data are fully reported within the study. Additional information can be requested from the corresponding authors.

## Abbreviations

The following abbreviations are used in this manuscript:

ACCF	Anterior cervical corpectomy and fusion
ACDF	Anterior cervical discectomy and fusion
AO Spine	Arbeitsgemeinschaft für Osteosynthesefragen Spine
CI	Confidence interval
DCM	Degenerative cervical myelopathy
DCM	Degenerative cervical spondylolisthesis
IQR	Interquartile range
LF	Ligamentum flavum
MRI	Magnetic resonance imaging
mJOA	modified Japanese Orthopaedic Association
OPLL	Ossification of the posterior longitudinal ligament
OR	Odds ratio
Post Dec	Posterior decompression alone
Post Dec + Fix	Posterior decompression with instrumented fixation
RECODE-DCM	Research Objectives and Common Data Elements for Degenerative Cervical Myelopathy
SCOR	Spinal cord occupation ratio
SD	Standard deviation
WFNS	World Federation of Neurosurgical Societies
